# Diagnostic Value of Serum Biomarkers for Differentiating Central and Peripheral Causes of Acute Vertigo

**DOI:** 10.3389/fmed.2020.00084

**Published:** 2020-03-19

**Authors:** Jong-Hee Sohn, Chul-Ho Kim, Sang-Hwa Lee, Jong Ho Kim, Jae Jun Lee

**Affiliations:** ^1^Department of Neurology, Chuncheon Sacred Heart Hospital, Hallym University College of Medicine, Chuncheon-si, South Korea; ^2^Department of Anesthesiology and Pain Medicine, Chuncheon Sacred Heart Hospital, Hallym University College of Medicine, Chuncheon-si, South Korea

**Keywords:** vertigo, central, peripheral, biomarker, NSE, S100β

## Abstract

**Background:** In patients presenting with acute vertigo or dizziness, distinguishing central from peripheral is a diagnostic challenge. This study investigated potential serum markers for differentiating central and peripheral vertigo in patients with acute-onset vertigo.

**Methods:** This prospective case–control study recruited consecutive participants from the Emergency Department, including patients with acute-onset vertigo or dizziness within 12 h and control subjects. We used enzyme-linked immunosorbent assays to measure the serum S100β, NSE, BDNF, GFAP, and IL-6 levels during the acute period.

**Results:** The 114 study subjects included 28 patients with central vertigo (CV), 49 patients with peripheral vertigo (PV), and 37 age- and sex-matched healthy controls. No differences were found in risk factor distribution among the three groups. In patients with CV, the serum NSE and S100β levels were significantly (*p* < 0.05) elevated compared with the control and PV groups. The ROC analysis gave an AUC of 0.843 (95% CI = 0.753–0.932) for NSE and 0.787 (95% CI = 0.687–0.886) for S100β for predicting CV. However, there were no significant differences in the serum GFAP and BDNF levels among the CV, PV, and control groups.

**Conclusions:** Serum NSE and S100β levels are significantly higher in patients with CV, such as occurs with posterior circulation ischemic stroke or vertebrobasilar insufficiency. S100β and NSE may serve as serum biomarkers for differentiating between CV and PV in patients with acute-onset vertigo.

## Introduction

Dizziness or vertigo is a major disease in the US that costs about $ 1.1 billion a year in medical expenses and accounts for 3.3% of the symptoms of patients visiting the emergency room (ER) (1, 2). Although vertigo is usually caused by diseases with clinically benign prognoses, such as peripheral causes, it also has central causes, such as cerebrovascular disease (2–5). Consequently, failure to determine the cause accurately can lead to serious complications.

Clinically, it is difficult to discriminate between peripheral and central causes. Patients presenting to the ER with dizziness as the main symptom require various examinations, including imaging examinations, to make a final diagnosis, which is time-consuming and expensive (1, 2, 6). Although diffusion-weighted magnetic resonance imaging (DWI) can detect early cerebral infarction, DWI is negative in the early stages of vertigo due to central causes, such as cerebral ischemia or vascular insufficiency, which can lead to errors in diagnosing central causes (7). Magnetic resonance imaging (MRI) is not available in some ERs and it is not cost effective to perform MRI in all patients with acute-onset dizziness or vertigo.

There are many limitations when it comes to evaluating patients with acute-onset dizziness in the ER. Recently, it has been reported that specialized tests for neuro-ophthalmologic disorders, such as head-thrust, gaze-evoked nystagmus, and skew deviation, are helpful in distinguishing central (CV) and peripheral (PV) vertigo (8–11). However, the sensitivity and specificity of these tests are low in the ER environment. Therefore, complementary tests are needed to distinguish between CV and PV.

The usefulness of blood biomarkers for differentiating CV and PV has been reported. Serum d-dimer, fibrinogen, and C-reactive protein were not useful for distinguishing between CV and PV (12). Serum S100 binding protein β (S100β) is somewhat useful for distinguishing CV from PV because of its association with the cause of CV, but it is not sensitive enough to determine the need for MRI in patients with acute vertigo (13).

Among brain-specific proteins, S100β, neuron-specific enolase (NSE) and glial fibrillary acidic protein (GFAP) have attracted increasing attention in clinical neurological research (14–19). S100β and GFAP, which is a marker of glial damage or activation, and NSE, which is a marker of neuronal damage, are proteins generally considered to be central nervous system (CNS) markers. Brain-derived neurotrophic factor (BDNF) is a protein found in the brain that belongs to the neurotrophin family of growth factors and is easily detected in peripheral blood (20–23). BDNF has central roles in brain development, physiology, and pathology. Aside from its importance in neural development and cell survival, BDNF appears essential for the molecular mechanisms of synaptic plasticity (20), and neuroprotective actions of BDNF were suggested in a rat model of transient forebrain ischemia (24). Interleukin-6 (IL-6), a major cytokine that stimulates the synthesis of acute-phase proteins, is regulated by the CNS, and is associated with several parameters of acute stroke (25, 26). This evidence supports the hypothesis that serum biomarkers can help to distinguish between CV and PV. In this study, to investigate potentially useful markers for distinguishing between central and peripheral causes of acute-onset vertigo, we compared the serum S100β, NSE, GFAP, BDNF, and IL-6 levels in patients with acute-onset vertigo and control subjects.

## Methods

This single-center, prospective, case-control study was performed at Chuncheon, Sacred Heart Hospitals of Hallym University College of Medicine from October 2017 to March 2019. The study protocol and informed consent form were reviewed and approved by the Institutional Review Board. Written informed consent was obtained from all participants before enrolling them in the study.

### Subjects

We enrolled patients with acute-onset vertigo and age- and sex-matched controls in a case–control design. Patients with a chief complaint of acute-onset vertigo or dizziness accompanied by nausea, headache, and unsteadiness who visited the ER within 12 h of symptom onset were eligible for the study. All participants were examined by a board-certified neurologist; this included the patient's history, a neurological examination, and neuroimaging studies. They underwent cerebral computed tomography (CT) and subsequent cerebral MRI with contrast-enhanced magnetic resonance angiography (MRA) to visualize the extracranial vessels. We recruited the control group via advertisements, such as through posted notices in the hospital. The control group consisted of volunteers who had no neurological symptoms and matched the patients in the CV group by age, sex, and vascular risk factors, including hypertension, diabetes mellitus, and smoking. Exclusion criteria were subjects with neurological disorders such as epilepsy, multiple sclerosis, or other known inflammatory CNS disorders, malignancies such as brain tumors and lung cancer, psychiatric illnesses, a history of head trauma ever, the presence of impaired renal function, age under 18 years, and pregnancy. The CV group had a cerebral infarct in the posterior circulation or vertebrobasilar insufficiency (VBI). Central causes of vertigo were defined as diffusion restriction changes in the posterior circulation or any transient neurological deficit presumed to be caused by a lesion in the vertebrobasilar territory without evidence of acute infarction, but occlusion or stenosis >50% in the vertebrobasilar arteries on MRA. The PV group had a definite diagnosis of either vestibular neuronitis or benign paroxysmal positional vertigo (BPPV) based on published criteria and the laboratory findings, including video-electronystagmography with the position test, caloric testing, and the head impulse test.

### Collection of Blood Samples and Biochemical Analysis

Blood samples from the patients with vertigo were obtained within 12 h of symptom onset. Plasma was centrifuged at 3,000 rpm for 10 min and serum was frozen within 30 min at −70°C until assayed. The serum S100β, NSE, BDNF, GFAP, and IL-6 levels were determined using enzyme-linked immunosorbent assay (ELISA) kits. All assays were performed exactly as described by the manufacturers. We used the following kits: Human S100β and GFAP ELISA Kits (Merck Millipore, CO, USA), and Human Enolase 2/Neuron-specific Enolase, Human Free BDNF, and Human IL-6 Quantikine ELISA Kits (R&D Systems, Minneapolis, MN, USA).

### Statistical Analysis

Statistical analyses were performed using the Statistical Packages for the Social Sciences for Windows ver. 22.0 (IBM, Armonk, NY, USA). To compare demographic and clinical parameters among groups, analysis of variance (ANOVA) and the chi-square test were used. We used a one-sided tests in the ANOVA and chi-square test analysis. After identifying the non-normal distribution of the serum biomarker levels using the Kolmogorov–Smirnov test, they were compared using the non-parametric Mann–Whitney *U*-test. We used a two-sided tests in the Mann-Whitney test. Differences between subgroups were analyzed using the Kruskal–Wallis test. Receiver-operating characteristics (ROC) analysis was performed to determine the sensitivity and specificity for distinguishing CV from other groups for each serum biomarker. A *p*-value < 0.05 was considered statistically significant.

## Results

### Clinical Characteristics of the Patients With Central vs. Peripheral Vertigo

The study included 114 subjects. Of the 77 patients with acute-onset vertigo, 28 had central causes (posterior circulation cerebral infarct or VBI) and 49 had peripheral causes (vestibular neuronitis or BPPV). The control group comprised 37 age- and sex-matched subjects. There were no significant differences in age (CV = 64.46 years, PV = 63.28 years, controls = 60.37 years), or the percentage of males, hypertension, diabetes mellitus, cardiac disease, and active smokers among the groups (Table 1).

**Table 1:** Subject characteristics.

	**Central vertigo** **(*n* = 28)**	**Peripheral vertigo** **(*n* = 49)**	**Controls** **(*n* = 37)**
Age (years)	64.46 ± 13.76	63.28 ± 12.45	60.37 ± 5.85
Male (*n*, %)	17 (60.7%)	27 (55.1%)	23 (62.2%)
Hypertension (*n*, %)	15 (53.6%)	24 (49.0%)	19 (51.4%)
Diabetes mellitus (*n*, %)	5 (17.9%)	10 (20.4%)	8 (21.5%)
Cardiac disease (*n*, %)	4 (14.3%)	7 (14.3%)	4 (10.8%)
Smoking (*n*, %)	5 (17.9%)	7 (14.3%)	6 (16/2%)

*Values are expressed as the mean ± standard deviation or number (%)*.

### Serum Biomarker Levels in Patients With Central vs. Peripheral Vertigo

**Figure 1:**
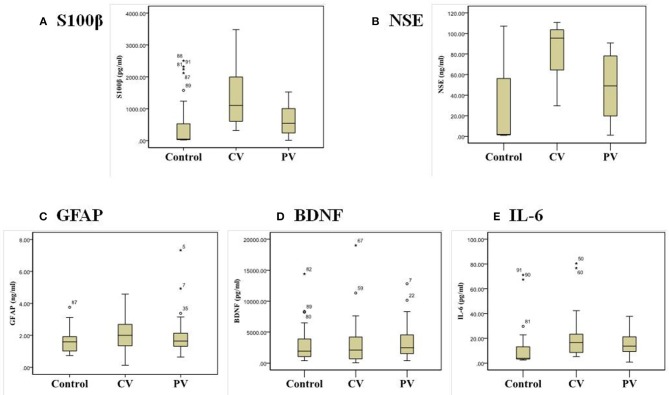
Box-and-whisker plots of the serum biomarker concentrations: **(A)** S100β, **(B)** NSE, **(C)** GFAP, **(D)** BDNF, and **(E)** IL-6 in patients with central and peripheral vertigo.

The CV group had increased serum S100β, NSE, and IL-6 levels, whereas the PV group had an increased serum IL-6 level compared with the controls (*p* < 0.05). In the CV group, the serum S100β [median 969.61 pg/mL, interquartile range (IQR) 538.02–2,023.36] and NSE (median 92.18 ng/mL, IQR 63.00–104.12) levels were significantly elevated compared with the PV group (S100β: median 347.30 pg/mL, IQR 242.36–1,029.10; NSE: median 19.81 ng/mL, IQR 17.62–49.08) (*p* < 0.05). But, there were no significant differences in the serum GFAP and BDNF levels among the three groups (Table 2). Figure 1 shows box-and-whisker plots of the serum concentrations of each biomarker among the groups. Of the 28 patients in the CV group, 17 had a posterior circulation cerebral infarct and 11 had a VBI. There were no significant differences in the serum biomarkers between these two patient subgroups.

**Table 2:** Differences in potential serum biomarkers between patients with central and peripheral vertigo.

**Serum biomarkers**	**Median, (inter-quartile range)**		
	**Central vertigo** **(*n* = 28)**	**Peripheral vertigo** **(*n* = 49)**	**Controls** **(*n* = 37)**
S100β (pg/mL)	969.61^*^^#^ (538.02–2,023.36)	347.30^#^ (242.36–1,029.10)	47.18^*^ (28.87–667.48)
GFAP (ng/mL)	1.07 (1.33–2.73)	1.54 (1.30–2.16)	1.58 (1.02–1.97)
BDNF (pg/mL)	1,788.68 (660.91–4,267.97)	1,879.51 (1,493.25–4,722.45)	1,916.24 (1,038.41–3,897.56)
NSE (ng/mL)	92.18^*^^#^ (63.00–104.12)	19.81^#^ (17.62–49.08)	1.90^*^ (1.39–59.04)
IL-6 (pg/mL)	16.51^*^ (7.73–23.74)	14.46^†^ (9.20–21.75)	4.49^*^^†^ (3.26–14.37)

*All values are expressed as the median and inter-quartile range*.

* *p < 0.05 between patients with central vertigo and controls*.

† *p < 0.05 between patients with peripheral vertigo and controls*.

# *p < 0.05 between patients with central and peripheral vertigo*.

*P-values were determined using the Mann–Whitney U-test*.

### Determination of the Specificity and Sensitivity by ROC Analysis

**Figure 2:**
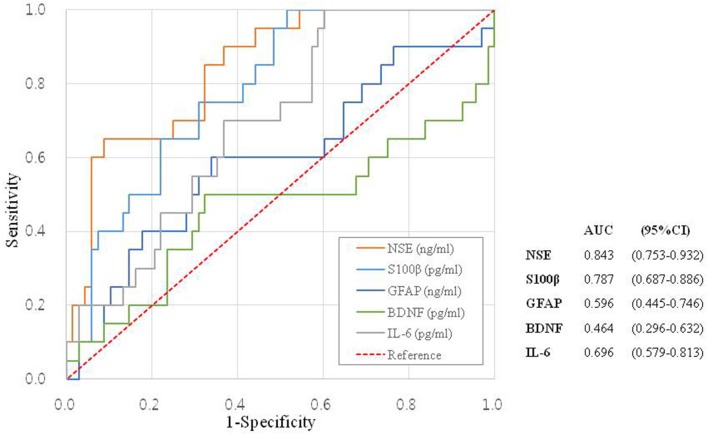
The ROC curve as a function of the specificity of each tested biomarker.

To determine the sensitivity and specificity of the biomarkers in patients with acute-onset vertigo, we created ROC curves for each serum biomarker (Figure 2). The ROC analysis gave an AUC of 0.843 (95% CI = 0.753–0.932) for NSE and 0.787 (95% CI = 0.687–0.886) for S100β, for predicting CV. NSE had a sensitivity of 70.0% and specificity of 70.6% for CV using a cut-off concentration of 73.1494 ng/mL. S100β had a sensitivity of 70.0% and specificity of 69.1% for CV using a cut-off concentration of 766.9938 ng/mL.

## Discussion

This study found that the serum NSE and S100β levels were significantly higher in the CV group (i.e., posterior circulation ischemic stroke or VBI) compared with the PV group and controls. No significant differences were found in the serum GFAP and BDNF levels among the three groups. Thus, NSE and S100β may serve as serum biomarkers for differentiating between central and peripheral causes in patients with acute-onset vertigo.

Vertigo/dizziness is a common reason for patients to present to the ER, accounting for 2.5% of ER visits during a 10-year period (27). The rate of visits for vertigo/dizziness is increasing as a percent of all visits to US ERs and the use of diagnostic tests including CT/MRI has increased dramatically (6). Distinguishing central causes from benign peripheral causes in patients with acute-onset vertigo is a diagnostic challenge. It is difficult to differentiate between the two causes at the bedside and no clinical test in isolation has been found to be reliable for distinguishing central from peripheral causes (9). A clinical examination of a patient with vertigo by a specialist can be a powerful tool for distinguishing between central and peripheral causes, such as a three-step bedside oculomotor examination (HINTS; Head Impulse-Nystagmus-Test of Skew) and quantitative video-oculography (10, 28). However, these tests may be limited by the availability of trained specialists and the low sensitivity and specificity of the test in the ER environment (29). Early DWI in acute posterior circulation stroke misses up to one in five strokes in the first 24–48 h (29–31). Thus, simple serum biomarkers can be especially helpful for patients with acute-onset vertigo if the causes of vertigo can be distinguished and the clinical outcome can be predicted.

Several studies of potential biomarkers in patients with vertigo have been reported. One study reported that serum d-dimer, fibrinogen, and C-reactive protein levels were not useful for differentiating central and peripheral vertigo (12). Another study found that serum S100β levels were associated with the presence of central causes of vertigo on brain MRI, although the levels were not sufficiently sensitive to exclude candidates for brain MRI (13). In addition, serum S100β levels were significantly higher in posterior circulation stroke patients than in nonvascular vertigo patients. Serum MMP-9 levels tended to be higher in stroke patients, whereas no significant differences among groups were found for sVCAM-1 and GFAP (32). Similar to previous results, we found that the serum S100β levels were significantly higher in CV patients, whereas no significant difference among groups was found for GFAP. Serum GFAP is a sensitive, specific biomarker of intracerebral hemorrhage in patients with acute stroke symptoms. The time window of between 1 and 6 h after stroke onset is best for using GFAP to differentiate between intracerebral hemorrhage and ischemic stroke (33–35). By contrast, in ischemic stroke, the structural integrity of brain cells and the blood–brain barrier are preserved for a longer time after symptom onset. Therefore, we found no difference in the serum GFAP levels between CV and PV patients because we excluded patients with intracerebral hemorrhage.

The main finding of this study was that of the significantly increased serum NSE and S100β levels in the CV group. Thus, NSE and S100β may serve as serum biomarkers for differentiating between central and peripheral causes in patients with acute-onset vertigo. S100β is considered to be a biomarker of CNS injury. This protein is a member of a family of Ca+-binding proteins and is abundant in the nervous system, mostly in astrocytes and several neuronal populations (36). Increased serum levels of this protein can be found in patients with cerebrovascular disease, cerebral trauma, or brain hypoxia due to cardiovascular arrest, but also in patients with schizophrenia, epilepsy, or Alzheimer's disease (37–41). NSE is a dimeric isoenzyme of the glycolytic enzyme enolase. It is localized mainly within neurons and cells of neuroendocrine origin and is considered to be a biomarker of neuronal loss (42). Many studies have reported elevated serum NSE concentrations in a variety of conditions associated with CNS damage, such as stroke, traumatic brain injury, Alzheimer's disease, and multiple sclerosis (43–46). Similar to our findings, increased serum NSE has been also reported to be the strongest indicator of acute cerebral infarct in patients presenting to the ER with acute isolated vertigo or dizziness (47).

In a subgroup analysis of our CV group, there was no significant difference in the serum biomarkers levels between posterior circulation cerebral infarct and VBI. VBI is defined by inadequate blood flow through the posterior circulation of the brain, supplied by the two vertebral arteries that merge to form the basilar artery. Therefore, it is often used to identify transient ischemic attacks (TIAs) in the vertebrobasilar territory. Although patients may initially be asymptomatic, a significant build-up of atherosclerosis plaque over time may lead to ischemic events. Therefore, VBI is an important diagnosis to consider, as many symptoms can mimic benign peripheral etiologies in the emergency setting. Therefore, in the VBI group, serum biomarker levels similar to those of cerebral infarct in the posterior circulation have important clinical implications. Both brain-specific proteins (NSE & S100β) were increased in hypertensive patients in previous investigations and the serum NSE concentration is a useful biomarker for predicting subclinical brain damage, such as white matter lesions and future vascular events related to the CNS (stroke, TIA, vascular headache, or migraine) in patients with hypertension (48, 49). These findings could be a starting point for future investigations of serum biomarkers for the detection of subclinical brain damage in VBI or stroke-prone patients, and constitute risk factors.

Interleukin-6 is widely produced in the initial phases of acute and chronic inflammation by fibroblasts, endothelial cells, macrophages, lymphocytes, and neutrophils. The serum IL-6 levels in the CV and PV groups were increased compared with the controls. A previous study investigated the roles of oxidative stress and inflammatory mediators in BPPV and vascular vertigo (50, 51). The levels of pro-inflammatory mediators, including IL-6, were higher in the BPPV group and decreased with a repositioning maneuver (50). The IL-6 levels in vascular vertigo were also significantly higher than those of non-vascular vertigo and controls (51). Therefore, the inflammatory mediator IL-6 may not be appropriate as a serum biomarker for distinguishing central and peripheral causes in patients with acute-onset vertigo. In BPPV patients, various inflammatory biomarkers, such as the neutrophil-to-lymphocyte ratio, platelet-to-lymphocyte ratio and mean platelet volume were significantly higher than in the controls (52). Recently, both the neutrophil count and neutrophil-to-lymphocyte ratio were found to have diagnostic value in distinguishing between acute cerebral infarct and vertigo (53). More research needs to examine the role of inflammatory biomarkers for distinguishing CV and PV.

This study is limited by the small number of subjects and it is necessary to validate our findings with a larger cohort. Future studies should examine the association between serum biomarkers and clinical outcomes in broader patient populations. We also used a single blood measurement, which may not reflect the actual patient status, given that repeated or multiple measurements over time and changes in the measurements may provide a more accurate picture of each biomarker. S100β protein has a very short half-life. The timeliness of sampling and processing may affect the resulting heterogeneity of the obtained S100β levels (13, 54). These biochemical biomarkers may contribute to assessment and management in the ER. However, there is also a need for sensitive, noninvasive, inexpensive neurobiological health biomarker bioassays in the ER, such as electroencephalogram-based psychobiological measures, rather than the more expensive and invasive biochemical biomarkers (55, 56).

In conclusion, this study suggests that the serum NSE and S100β concentrations could be useful markers for distinguishing vertigo of central causes, such as a posterior circulation cerebral infarct or VBI, from peripheral causes and controls in the emergency setting. However, a larger sample size, longer follow-up period, and correlation of clinical outcomes are required. Further research is necessary to evaluate the role of potential biomarkers, such as S100β, NSE, GFAP, BDNF, and IL-6, in patients with acute-onset vertigo.

## Data Availability Statement

The datasets generated for this study are available on request to the corresponding author.

## Ethics Statement

The studies involving human participants were reviewed and approved by Chuncheon, Sacred Heart Hospitals of Hallym University College of Medicine, Institutional Review Board. The patients/participants provided their written informed consent to participate in this study.

## Author Contributions

J-HS and JL: conception and design of the work and writing the manuscript. J-HS, C-HK, and S-HL: acquisition of data for the work. J-HS, C-HK, S-HL, and JK: analysis and interpretation of data for the work.

### Conflict of Interest

The authors declare that the research was conducted in the absence of any commercial or financial relationships that could be construed as a potential conflict of interest.

## Abbreviations

CV: central vertigo
PV: peripheral vertigo
S100β: S100 binding protein B
NSE: Neuron-specific enolase
GFAP: Glial fibrillary acidic protein
BDNF: Brain-derived neurotrophic factor
IL-6: interleukin-6
DWI: Diffusion-weighted magnetic resonance imaging
MRI: magnetic resonance imaging
CT: computed tomography
MRA: magnetic resonance angiography
VBI: vertebrobasilar insufficiency
BPPV: benign paroxysmal positional vertigo
TIA: transient ischemic attack
ELISA: enzyme-linked immunosorbent assay.
